# Supplementary material for: Topological, Functional, and Phylogenetic Analysis of the Cell Division Site Selection Protein MinJ of Bacillus subtilis
Source: Mol Microbiol. 2026 Oct 12;126(5):596–612. doi: 10.1111/mmi.70119 (PMC13725322; doi:10.1111/mmi.70119)
Supplement: Supplementary file 1 [file MMI-126-596-s004.pdf]

Figure S1

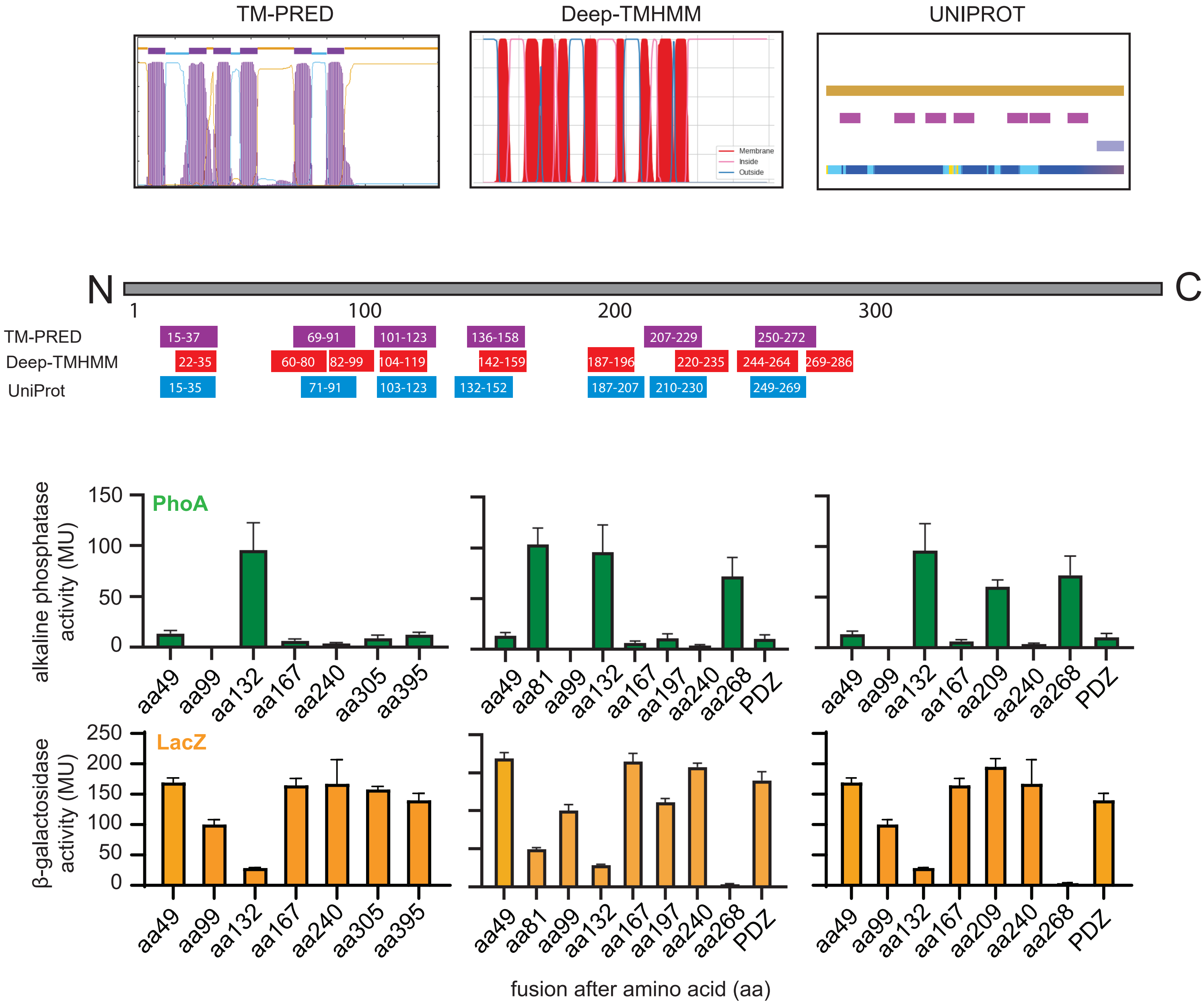
TM-PRED
Deep-TMHMM
UNIPROT
Membrane
Inside
Outside
N
C
1
100
200
300
TM-PRED
15-37
69-91
101-123
136-158
207-229
250-272
Deep-TMHMM
22-35
60-80
82-99
104-119
142-159
187-196
220-235
244-264
269-286
UniProt
15-35
71-91
103-123
132-152
187-207
210-230
249-269
PhoA
alkaline phosphatase activity (MU)
150
100
50
0
aa49 aa99 aa132 aa167 aa240 aa305 aa395
aa49 aa81 aa99 aa132 aa167 aa197 aa240 aa268 PDZ
aa49 aa99 aa132 aa167 aa209 aa240 aa268 PDZ
LacZ
β-galactosidase activity (MU)
250
200
150
100
50
0
aa49 aa99 aa132 aa167 aa240 aa305 aa395
aa49 aa81 aa99 aa132 aa167 aa197 aa240 aa268 PDZ
aa49 aa99 aa132 aa167 aa209 aa240 aa268 PDZ
fusion after amino acid (aa)

Figure S2

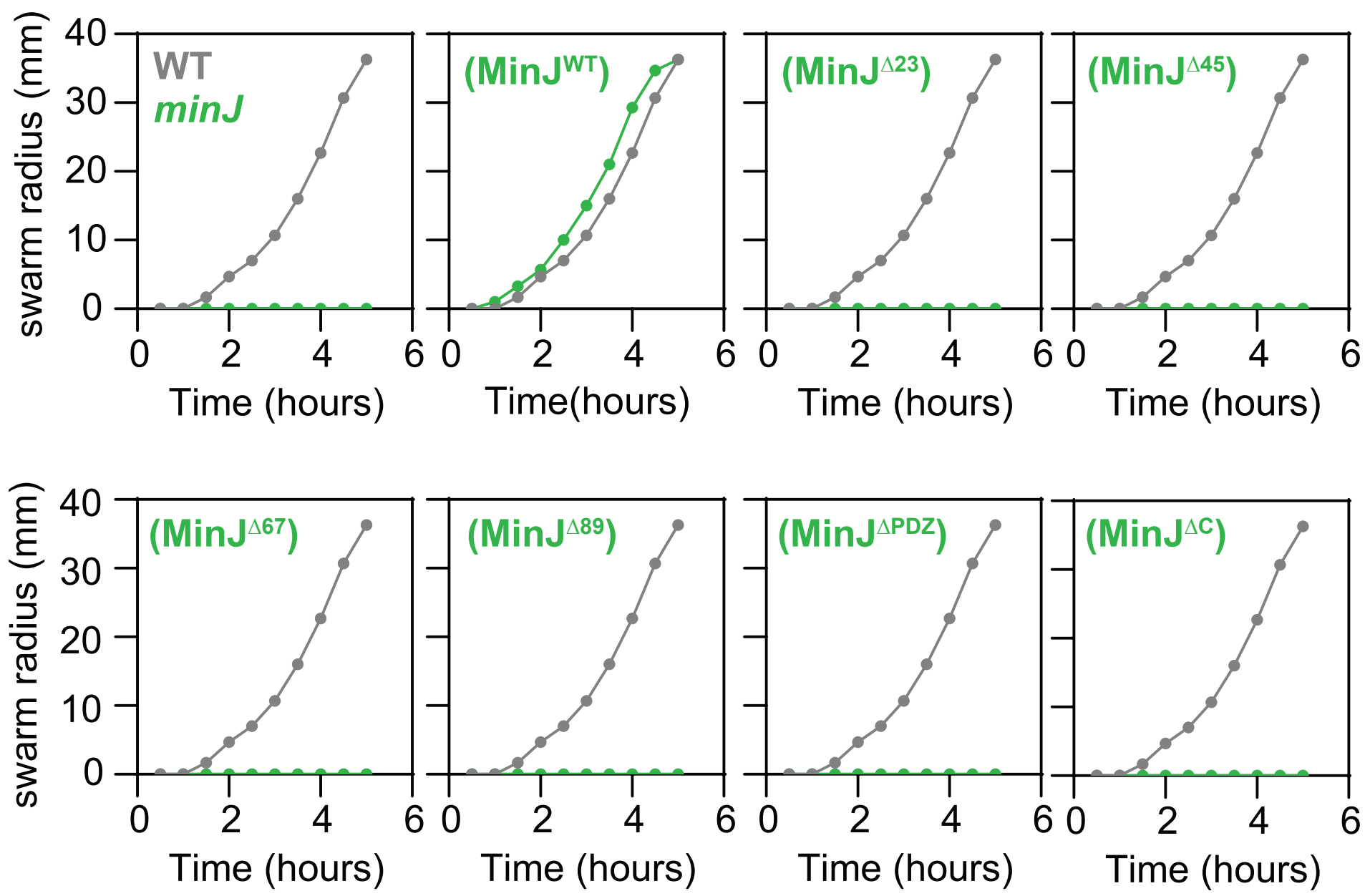
WT
minJ
(MinJWT)
(MinJΔ23)
(MinJΔ45)
(MinJΔ67)
(MinJΔ89)
(MinJΔPDZ)
(MinJΔC)
swarm radius (mm)
Time (hours)

Figure S3

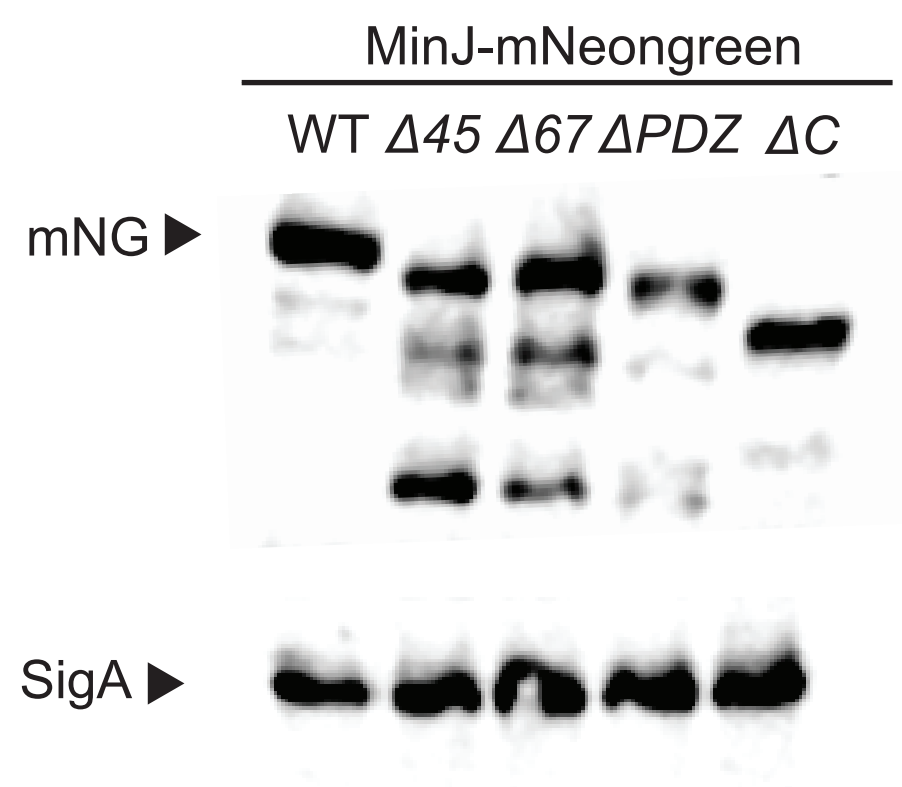

Figure S4

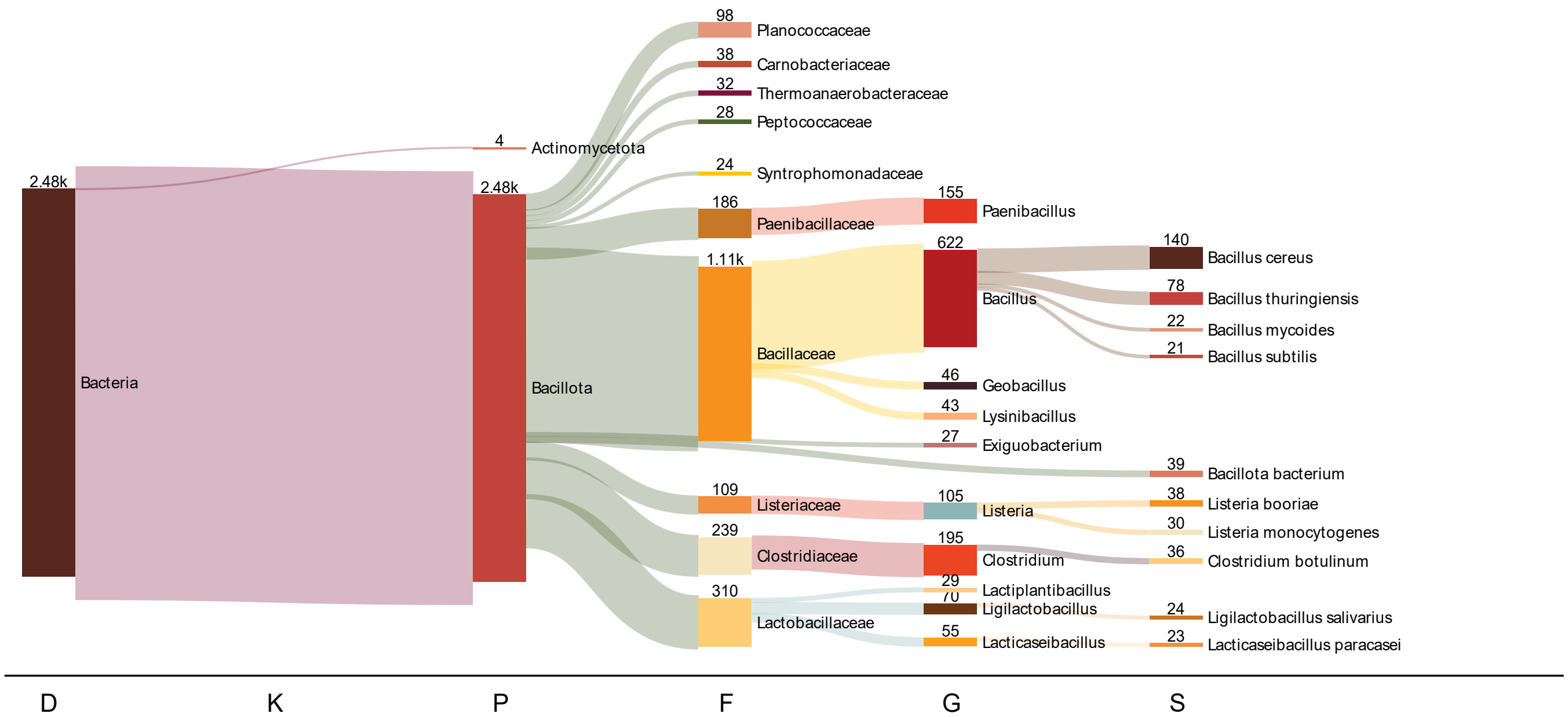
2.48k
Bacteria
4
Actinomycetota
2.48k
Bacillota
98
Planococcaceae
38
Carnobacteriaceae
32
Thermoanaerobacteraceae
28
Peptococcaceae
24
Syntrophomonadaceae
186
Paenibacillaceae
1.11k
Bacillaceae
109
Listeriaceae
239
Clostridiaceae
310
Lactobacillaceae
155
Paenibacillus
622
Bacillus
46
Geobacillus
43
Lysinibacillus
27
Exiguobacterium
105
Listeria
195
Clostridium
29
Lactiplantibacillus
70
Ligilactobacillus
55
Lacticaseibacillus
140
Bacillus cereus
78
Bacillus thuringiensis
22
Bacillus mycoides
21
Bacillus subtilis
39
Bacillota bacterium
38
Listeria booriae
30
Listeria monocytogenes
36
Clostridium botulinum
24
Ligilactobacillus salivarius
23
Lacticaseibacillus paracasei
D
K
P
F
G
S
