# Supplementary material for: Topological, Functional, and Phylogenetic Analysis of the Cell Division Site Selection Protein MinJ of Bacillus subtilis
Source: Mol Microbiol. 2026 Oct 12;126(5):596–612. doi: 10.1111/mmi.70119 (PMC13725322; doi:10.1111/mmi.70119)
Supplement: Supplementary file 2 [file MMI-126-596-s001.docx]

**Table S1 – Conserved residues across MinJ sequence^a^**

| Residue Position | Residue | Percent Conserved (%) |
| --- | --- | --- |
| 20 | P | 90.68 |
| 70 | S | 93.20 |
| 77 | G | 91.44 |
| 168 | P | 97.48 |
| 191 | P | 96.73 |
| 209 | P | 91.94 |
| 228 | G | 96.22 |
| 305 | G | 96.47 |
| 325 | G | 96.47 |
| 332 | N | 96.22 |
| 380 | G | 98.99 |

^a^Residues with greater than 90% conservation identity
