# Supplementary material for: Topological, Functional, and Phylogenetic Analysis of the Cell Division Site Selection Protein MinJ of Bacillus subtilis
Source: Mol Microbiol. 2026 Oct 12;126(5):596–612. doi: 10.1111/mmi.70119 (PMC13725322; doi:10.1111/mmi.70119)
Supplement: Supplementary file 3 [file MMI-126-596-s002.docx]

**Table S2 – Conserved sequence stretch across MinJ sequence^a^**

| Residue position | Length | Consensus | Conservation |
| --- | --- | --- | --- |
| 207-209 | 3 | WWP | 81.11% |
| 206-209 | 4 | PWWP | 73.43% |
| 353-357 | 5 | CKLEV | 59.80% |

^a^Sequence stretches of three or more with greater than 50% conservation identity
