# Supplementary material for: Topological, Functional, and Phylogenetic Analysis of the Cell Division Site Selection Protein MinJ of Bacillus subtilis
Source: Mol Microbiol. 2026 Oct 12;126(5):596–612. doi: 10.1111/mmi.70119 (PMC13725322; doi:10.1111/mmi.70119)
Supplement: Supplementary file 4 [file MMI-126-596-s003.docx]

**Table S4 – Primers**

| Primer | Sequences |
| --- | --- |
| 7642 | AGGAGGTCGACCTTGAGGCGGTGAGAGATAGTG |
| 7643 | CTCCTGCTAGCCGTTTCAGGCAGCGATCCCTG |
| 7644 | CTCCTGCTAGCGCCCTGATCCCTTTTCGAGAAATA |
| 7675 | CTCCTGCTAGCTGATGTTCTCACATGCGC |
| 7676 | CTCCTGCTAGCAAATCTCTCCAGAAAGGGCTC |
| 7677 | CTCCTGCTAGCAAGAGTGAACGCTGCCGC |
| 7678 | CTCCTGCTAGCTGCTATTCTTGTATGAAAGGTTTTTCG |
| 7712 | CTCCTGCTAGCGGGCCACCAGCTAAGGTG |
| 7713 | CTCCTGCTAGCCGGCGTCCACAGTAAGCT |
| 7729 | CTCCTGCTAGCAGGAATCGATATTCCGAGTCC |
| 7730 | CTCCTGCTAGCGGGCACGAGAAGGAACAG |
| 7731 | CTCCTGCTAGCTCCCGAAGCGACTGCTTC |
| 7906 | AGGAGGTCGACTTGAGGCGGTGAGAGATAGTG |
| 7907 | CTCCTGCTAGCTTACGCGTTATCATTCACTCTTTGCTT |
| 7956 | AGGAGGGATCCTATTTCTCGAAAAGGGATCAGGGC |
| 7957 | CTCCTGAATTCTGATCCCGAAGCGACTGC |
| 8399 | GTGAGCGGATAACAATTAAGCTTAC |
| 8400 | GAATTAGCTTGCATGCGTAACATCAGAG |
| 8468 | TTCAGGCAGCGATCCCTGTGTTCTCACATGCGCAGAACG |
| 8469 | CGTTCTGCGCATGTGAGAACACAGGGATCGCTGCCTGAA |
| 8470 | ATTTATGATGATCTGAAATTTACATATACACGCGCAAACTGGATGTCTGCT |
| 8471 | AGCAGACATCCAGTTTGCGCGTGTATATGTAAATTTCAGATCATCATAAATATCTGC |
| 8472 | GCAGCGTTCACTCTTCGCGCGCATGTGAGAACATCA |
| 8473 | TGATGTTCTCACATGCGCGCGAAGAGTGAACGCTGC |
| 8476 | AGGATCACAGCGAAACGCTATTTCTCGAAAAGGGATCAGGGC |
| 8477 | GCCCTGATCCCTTTTCGAGAAATAGCGTTTCGCTGTGATCCT |
| 8478 | AATGATAACGCGGCTCCGTTTGGAGAAATTCGTTTTGACCAG |
| 8479 | CTGGTCAAAACGAATTTCTCCAAACGGAGCCGCGTTATCATT |
| 8635 | GCCGCTGAAGCTTCAGCTGAGTAACGTTAGCGAGAATGC |
| 8636 | AATTCCCCATTGAACAGACACTATCTCTCACCGCCTCAA |
| 8637 | TTGAGGCGGTGAGAGATAGTGTCTGTTCAATGGGGAATT |
| 8638 | CATGCGAGTCGACTTGCTTTATGATCCCGAAGCGACTGC |
| 8818 | CTCCTGAATTCCCGCCAGAACCAGCAGCGGAGCCAGCGGAACCGACTGCTTCGTCTTCACGGTCAT |
| 8819 | AGGAGGGATCCGTCTGTTCAATGGGGAATTGAACTG |
| 8844 | AGGAGGTCGACGGATCCGCTGGGCTCCGCTGCTGGTTCTGGCGCTAGCATGGTTTCGAAAGGAGAGGAG |
| 8845 | GAATTCTTACTTATAGAGTTCATCCATACCCAT |
| 8846 | CTTCAGCTAGCAAGTCGACGTTGAAGAGGGCAAGTATTGTG |
| 8847 | GCAGCGGAGCCCAGCGGATCTGATCCCGAAGCGACTGC |
| 9166 | GAATTCCCGCCAGAACCAGCAGCGGAGCCAGCGGAACCCGCGTTATCATTCACTCTTTGCTT |
