# Supplementary material for: Topological, Functional, and Phylogenetic Analysis of the Cell Division Site Selection Protein MinJ of Bacillus subtilis
Source: Mol Microbiol. 2026 Oct 12;126(5):596–612. doi: 10.1111/mmi.70119 (PMC13725322; doi:10.1111/mmi.70119)
Supplement: Supplementary file 5 [file MMI-126-596-s005.docx]

**Table S3 – Plasmids**

| Plasmids | Genotype |
| --- | --- |
| pJP31 | *minD -linker-cyaAT25 kan* |
| pJP42 | *divIVA -linker-cyaAT25 kan* |
| pKO2 | *amyE::P_hyspank_ minJ^aa240^-lacZ spec amp* |
| pKO3 | *amyE::P_hyspank_ minJ^aa240^-phoA spec amp* |
| pKO4 | *amyE::P_hyspank_ minJ^aa305^-lacZ spec amp* |
| pKO5 | *amyE::P_hyspank_ minJ^aa305^-phoA spec amp* |
| pKO8 | *amyE::P_hyspank_ minJ^aa167^-lacZ spec amp* |
| pKO9 | *amyE::P_hyspank_ minJ^aa132^-lacZ spec amp* |
| pKO10 | *amyE::P_hyspank_ minJ^aa99^-lacZ spec amp* |
| pKO11 | *amyE::P_hyspank_ minJ^aa49^-lacZ spec amp* |
| pKO12 | *amyE::P_hyspank_ minJ^aa167^-phoA spec amp* |
| pKO13 | *amyE::P_hyspank_ minJ^aa132^-phoA spec amp* |
| pKO14 | *amyE::P_hyspank_ minJ^aa99^-phoA spec amp* |
| pKO15 | *amyE::P_hyspank_ minJ^aa49^-phoA spec amp* |
| pKO18 | *amyE::P_hyspank_ minJ^aa209^-lacZ spec amp* |
| pKO19 | *amyE::P_hyspank_ minJ^aa268^-lacZ spec amp* |
| pKO20 | *amyE::P_hyspank_ minJ^aa209^-phoA spec amp* |
| pKO21 | *amyE::P_hyspank_ minJ^aa268^-phoA spec amp* |
| pKO25 | *amyE::P_hyspank_ minJ^aa81^-lacZ spec amp* |
| pKO26 | *amyE::P_hyspank_ minJ^aa81^-phoA spec amp* |
| pKO27 | *amyE::P_hyspank_ minJ^aa197^-lacZ spec amp* |
| pKO28 | *amyE::P_hyspank_ minJ^aa197^-phoA spec amp* |
| pKO29 | *amyE::P_hyspank_ minJ^aaPDZ^-lacZ spec amp* |
| pKO30 | *amyE::P_hyspank_ minJ^aaPDZ^-phoA spec amp* |
| pKO37 | *GST-PDZ amp* |
| pKO71 | *minJ-linker-cyaAT25 kan* |
| pKO72 | *amyE::P_minJ_-minJ amp spec* |
| pKO73 | *amyE::P_minJ_-minJ* ^ΔCterm^ *amp spec* |
| pKO76 | *amyE::P_minJ_-minJ* ^Δ45^ *amp spec* |
| pKO79 | *amyE::P_minJ_-minJ* ^ΔPDZ^ *amp spec* |
| pKO103 | *amyE::linker-mNeongreen spec amp* |
| pKO106 | *minJ-linker-cyaAT18 amp* |
| pKO110 | *amyE::P_minJ_-minJ-linker-mNeongreen amp spec* |
| pKO115 | *amyE::P_minJ_-minJ* ^Δ67^*-linker-mNeongreen amp spec* |
| pKO116 | *amyE::P_minJ_-minJ* ^ΔPDZ^*-linker-mNeongreen amp spec* |
| pKO123 | *amyE::P_minJ_-minJ* ^Δ67^ *amp spec* |
| pKO125 | *amyE::P_minJ_-minJ* ^Δtail^*-linker-mNeongreen amp spec* |
| pKO127 | *amyE::P_minJ_-minJ* ^Δ45^*-linker-mNeongreen amp spec* |
| pKO136 | *minJ* ^ΔPDZ^ *-linker-cyaAT25 kan* |
| pKO143 | *thrC::P_hyspank_-mCherry amp mls* |
| pKO146 | *minJ* ^ΔPDZ^ *-linker-cyaAT18 amp* |
| pKO147 | *minJ* ^Δ67^ *-linker-cyaAT18 amp* |
| pKO148 | *minJ* ^Δ45^ *-linker-cyaAT25 kan* |
| pKO149 | *minJ* ^Δ67^ *-linker-cyaAT25 kan* |
| pKO151 | *minJ* ^Δ45^ *-linker-cyaAT18 amp* |
| pKO155 | *minJ* ^ΔCterm^ *-linker-cyaAT25 kan* |
| pKO157 | *minJ* ^ΔCterm^ *-linker-cyaAT18 amp* |
